# North-eastern Nigeria: assessing the response capacity of National Emergency Management Agency to the plights of internally displaced persons

**DOI:** 10.1016/j.heliyon.2021.e07274

**Published:** 2021-06-09

**Authors:** Shittu Raji, Folashade Arinola Adekayaoja, Emmanuel Ayila Agaku, James Akujobi, Ade Ayinde Hamzat

**Affiliations:** Centre for Peace and Strategic Studies, University of Ilorin, Nigeria

**Keywords:** Plights, Displacement, Intervention, NEMA

## Abstract

The plights of Internally Displaced Persons (IDPs), including hunger, rape, insecurity, and death, have assumed a frightening dimension in North-eastern Nigeria with the sustained intervention of the National Emergency Management Agency (NEMA) to ameliorate their sufferings. This paper aimed to assess the response capacity of NEMA to the plights of the IDPs in North-eastern Nigeria. The objectives of the study were to examine the nature and dimensions of the plights of the IDPs in the Northeast, identify their coping strategies, and assess the impact of NEMA's response strategies on the burgeoning situation. The study used a qualitative survey research design, in which 166 key Informant Interviews (KII) were conducted. The primary designs were complemented by secondary data, which was derived from books, journal articles, and News tabloids. Data generated were descriptively analyzed. Major findings from the paper were that the Boko Haram insurgency was primarily responsible for the mass displacement of persons in North-eastern Nigeria. The IDPs are facing lots of challenges in the camps with weak coping strategies while the intervention of NEMA through the provision of relief materials, rehabilitation and resettlement programs, and linking of the displaced with family members have reduced the plights of the IDPs. However, the Agency currently has the minimal operational capacity to ameliorate the plights of the IDPs due to the protracted nature of the insurgency, legal restriction on the operational mandates of NEMA, corruption, and limited funding of the agency. The Study recommended improved funding of the Agency and enactment of IDP-specific constitutional roles for maximum impact of NEMA on the amelioration of the plights of the IDPs in North-Eastern Nigeria.

## 1 Introduction

The plights of displaced persons across the world have become a formidable global challenge with overwhelming disastrous implications for human development, including hunger, insecurity, and death, among other growing humanitarian crises (International Displacement Monitoring Centre, 2019). One of the greatest direct consequences of such displacement is the upsurge of Internally Displaced Persons (IDPs) (International Displacement Monitoring Centre, 2019).

Since 2009, North-eastern Nigeria, which consists of six states that include Borno, Adamawa, Yobe, Bauchi, Gombe, and Taraba has largely been challenged by the internal displacement crisis. Mba (2017) finds that 13.33% of the IDPs in the area were displaced by communal clashes, 0.99% by natural disasters and 85.68% of the displacement was caused by the Boko Haram insurgency. The above statistics confirm that insurgency has largely been responsible for the mass internal displacement of persons, especially in Borno, Adamawa, and Yobe states, which have remained the epicenter of insurgent activities in North-eastern Nigeria since 2009. The crisis, which is linked to poor governance and religious extremism, is characterized by bombing, kidnapping, and destruction of lives and property. It is on record that the Boko Haram insurgency had killed over 30,000 people between 2009 and 2018, driven over 1.8 million out of their homes, and destroyed property worth #16 billion (International Review of the Red Cross, 2018). Apart from the mass internal displacement of persons, the insurgency has also generated 177,000 refugees in neighboring countries of Cameroon, Chad, and the Niger Republic (Reliefweb, 2020).

Thus, the North-eastern States have witnessed the unprecedented negative impact of the Boko Haram insurgency from 2009 to date; which eventually culminated in the declaration of a State of Emergency (SOE) in the three most affected states of Borno, Yobe, and the Adamawa States in 2013 by Former President Goodluck Jonathan. Most of the 9.6 million people (National Bureau of Statistics, 2018) living in the then SOE states have been affected in one way or the other by the insurgency attacks, which have led to the collapse of the survival structures of many families, virtual extermination of communities, destruction of social infrastructure and intense destruction of supply chain process of the economy and dispersal of a larger chunk of citizens in the affected states.

Much of the physical destructions by the insurgents were recorded in Borno State as many Local Government Areas, including but not limited to Dikwa, Marte, Bama, Gamboru, Ngala, Gwoza were overrun. Other Local Government Areas that were attacked by the insurgents include Chibok, Askira Uba, Kala Balge, Kukawa, and Abadan (Reliefweb, 2020). The loss of lives, destruction of property, and the disruption of economic activities forced residents of these areas to flee to Maiduguri metropolis where they were crammed together in camps as Internally Displaced Persons (IDPs) while others squat amongst many poor host families in the city under precarious conditions devoid of privacy, basic hygiene and conveniences. In Yobe State, Gujba and Gulani Local Government Areas were at a point effectively taken over and cut off from Damaturu; the State capital with the destruction of the Katarko Bridge by the insurgents. Schools, public buildings, places of worship, markets, and residential buildings were also destroyed by the insurgents in the two Local Government Areas. During the peak of the crisis, the southwards expansion of the Boko Haram activities culminated in the invasion of Madagali and Michika Local Government Areas of Adamawa State. Mubi, the capital of the Mubi Local Government Area and the second-largest commercial town in the State was temporarily overrun and occupied by the insurgents. The palpable fear led to a mass exodus of IDPs into Yola; the Adamawa State capital where they took refuge in seven camps while others reside within the host communities. The insurgent threat also led to the forced closure of the Adamawa State University, Mubi (Samuel, 2020).

The National Emergency Management Agency (NEMA) has primarily been responsible for the coordination and curtailment of the plights of the IDPs in North-eastern Nigeria since 2009 (National Emergency Management Agency, 2018). The agency was charged with the primary responsibilities of formulating and implementing federal government policies on activities relating to disaster management in Nigeria, including the management of the IDPs crisis, and the coordination of programs of actions for efficient implementation of the resettlement programs, and security for the IDPs (National Emergency Management Agency, 2018). This study examines the plights of the IDPs in North-eastern Nigeria and the response capacity of the National Emergency Agency (NEMA).

## 2 Statement of the problem

The crisis of existence and challenges of survival confronting internally displaced persons in North-eastern Nigeria have assumed a disturbing phenomenon with protracted implications for the displaced (Anthony and Nwobashi, 2016, International Committee of the Red Cross (ICRC), 2016, Mba, 2017, International Committee of the Red Cross, 2018, Displacement, Tracking Matrix (DTM), 2020, Reliefweb, 2020). As reported by the International Review of Red Cross (2018), there are more than 2,000,000 displaced persons across the Northeast along 120,000 Nigerian displaced refugees from the area to the neighboring countries of Cameroon, Chad, and Niger in 2018. The DTM (2020) Round 33 Report put the number of internally displaced persons in the Northeast states of Borno, Yobe, Adamawa, Gombe, Bauchi, and Taraba at 2, 118,550 cutting across 436,058 households as of August 2020. Aside from sudden displacement, which is enough physical torture for most displaced persons from their communities in North-eastern Nigeria, the displaced are more vulnerable in the camp to further threats of shortages of essential items, insecurity, psychological depression, and low self-esteem, among others. Many stakeholders, including the federal and state governments along with local and international donor agencies, have made concerted efforts to address the plights of the IDPs within the available resources through several measures, including the provision of food and non-food items (DTM, 2020). Despite their efforts, intense suffering persists in the IDP camp in North-eastern Nigeria.

Though NEMA has intervened as the primary responder to the plights of the IDPs with the provision of relief materials, including food, accommodation, security, and resettlement packages, the present living condition of the IDPs in the North-east is still very worrisome; raising the assumption that NEMA is also bedeviled by its internal responder challenges to respond adequately to the plight of IDPs. This study aims to examine the plights of the IDPs in North-eastern Nigeria and assess the response capacity of NEMA to the bourgeoning crisis. The objectives of the study are to examine the nature and dimensions of the plights of the IDPs in the Northeast, identify their coping strategies, and assess the impact of NEMA's response strategies on the burgeoning situation from the legal, operational, coordinating, and financial frameworks. Important questions to be answered in this research include: What are the nature and dimensions of the IDPs in North-eastern Nigeria? What are their coping strategies and what is the response capacity of NEMA to the plights of the IDPs in North-eastern Nigeria?

An important policy and academic gap, which this study intends to bridge is the lack of adequate study on NEMA's operational capacity to adequately respond to the needs of the IDPs despite the primary relevance of the agency to the welfares of the displaced. In the first instance, NEMA is the only federal government agency saddled with the coordinating responsibilities of taking care of the IDPs along with other tasks on disaster management. The agency also coordinates the activities of other responders to the plights of the IDPs, including the security agencies, state emergency management agencies, and international donor agencies while NEMA also sources for the funding of the IDPs. Despite the above central roles in the direct and indirect management of the IDPs, no critical studies have been carried out to examine the response capacity of NEMA to the plights of the IDPs in North-eastern Nigeria, a gap this study intends to bridge.

The study is also significant as it brings to the fore the imperative needs to address the prevailing difficulties being faced by the IDPs in North-Eastern Nigeria and the operational strength of NEMA at doing so by unveiling the agency's current achievements, operational gaps, and the challenges in meeting the needs of the IDPs. The study will also provide actionable recommendations to policymakers on ways of improving the capacity of NEMA in the management of the IDPs to improve their standard of living. This study will add to the body of knowledge as reference material on IDPs and disaster management.

## 3 Literature review

There are many scholarly works on the phenomenon of displacement and its impacts on the IDPs. As established in his study, Smith (2002) posits that displacement is one of the most tragic experiences for the displaced persons, which disrupts their socio-economic stability through loss of homes, jobs, and family members. The author identifies five categories of displaced people with one form of intense suffering or the other. They include those displaced outside their countries (Refugees) and individuals who sought international protection and whose claims for refugee status have not yet been determined (Asylum Seekers). Other categories of displacement are those who are displaced within a country (IDPs) and those who are former refugees that have returned voluntarily to their country but are still unable to return to their habitual homes due to devastation and insecurity (internally displaced ex-Refugees). Another category of displacement according to Smith (2002) is Stateless persons who are not considered as nationals of any state under international laws, especially those who formally possess a nationality which becomes ineffective due to various reasons.

Ramswamy (2008) identifies three critical sources of human displacement, which include war, natural calamities; including flood and earthquake, and development-induced displacement. The author also categorized natural disasters into three sub-dimensions, which include sudden and slow displacement and epidemic diseases-induced. Sudden disaster displacement is caused by obstructive and unanticipated disasters such as massive floods, major earthquakes, tsunami, volcanic eruptions, and landslides. These calamities usually lead to large-scale human displacement. Many times, noted by Hussain (2008), the economic crisis also displaces people from one place to another. The onset of economic displacement is usually a slow but steady process where young people leave home in search of jobs and settle down in the areas where they have access to a good job while epidemic diseases like Malaria and Cholera also displace people across the world. In his work, Imasuen (2014) finds that insurgency has become a primary threat to global peace and security; it is also the main source of internal displacement of persons, refugee debacles, and national security breaches. The author submits further that the impact of insurgency is felt in different parts of the world, including Africa because the destructive act has grown both in strength and trend.

Ajijola (2017) establishes the relationship between the Boko Haram insurgency and internal displacement in Nigeria by noting that since the insurgents began their campaign of terror against the Nigerian state, especially since 2009, several people across North-eastern Nigeria have been forced to flee their homes to safer places, the outcome of which is an unprecedented humanitarian crisis for the country. The author observes further that apart from being displaced physically from their ancestral homes, IDPs have also been displaced psychologically, emotionally, and socio-economically, which often leads to traumatic distress for the victims. Badurdeen (2010) recommended improved respect for international humanitarian law and promotion of good governance to reduce conflict and environmental disasters, which are the primary causes of internal displacement rather than establishing new laws for the protection of the IDPs.

The Assessment Capacity Project (ACP) (2018) identifies eight potential risks that every displaced person faces. These include landlessness, which removes the main foundation upon which the displaced productive capacity, commercial activities, and livelihoods are dependant, especially in the rural area where the livelihood of many residents is based on the agricultural produce; thus reducing their chance of tension-free life. Joblessness is another fundamental feature of displacement as job loss is very high among the displaced population. Job loss among the IDPs is essentially noticeable in both the rural and urban sectors, especially among those who are self-employed as agricultural laborers or industrial workers. Loss of shelter or homelessness is a major feature of displacement, such loss of a family's home along with their cultural heritage often results in alienation and social status deprivation. Forced displacement leads to loss of access to common property resources, including pastures, forested lands, water bodies, quarries, and agricultural land, community schools, which serve common and collective purposes for the displaced in their natural habitats. As posited by the Assessment Projects Capacity (2018), forced displacement is bound to cause food insecurity among the victims in form of reduced food availability, accessibility, affordability, and utility, which the ousters often face, leading to the food crisis, malnutrition, and undernourishment among the displaced persons.

According to Badurdeen (2010), there are concerted efforts at protecting internally displaced persons by the global community through the development of thirty Guiding Principles for Internal Displacement of Persons (GPID), which were designed and adopted by the United Nations to meet the challenges of severe deprivation, hardship, and discrimination encountered by the IDPs. The policies recognize the right of IDPs to be protected, defended, fed, and empowered while under the custody of the national authorities, and the non-state actors also have the moral responsibilities to care for the IDPs.

Related to the UN international Guiding Principles for the IDPs from the regional perspective is the African Union Convention on the Internally Displaced Persons (IDPs), known as the Kampala Convention (KC), which legally emphasizes the right of dignified existence of all IDPs within the country and the non-denial of their fundamental rights to dignity (International Displacement Monitoring Centre, 2019). Regrettably, out of the fifty-four member states of the African Union, only seven have laws and policies relating to IDPs. These include Angola, Burundi, Kenya, Liberia, Sierra Leone, Sudan, and Uganda (International Displacement Monitoring Centre, 2019). As noted by the International Displacement Monitoring Centre (2019), precisely, Nigeria does not have specific laws to cater to the wellbeing of the IDPs. Though a legal framework was proposed in 2006, the drafted bill was not passed into law. Nevertheless the GPID is non-binding on membership of the UN, it has gained considerable authority as a valuable practical guide to all stakeholders and responders to the plights of the IDPs globally.

In their work, Adesote et al. (2015) traced the genesis of internal displacement in Nigeria to the Biafran war (1967–1970), in which about 500,000 people died with about 1million others becoming internally displaced. Even though displacement of this magnitude has not been repeated in the country, there had been internal displacement in Nigeria in 2002 when approximately 30,000 people were forced to flee their homes after ethno-religious violence rocked the Northern states of Kaduna, Kano, Bauchi, Taraba, Nassarawa, Benue, and Plateau states, among others. According to the Global Report on Internal Displacement (2019), the exact human displacement figure in Nigeria is difficult to estimate because many internally displaced persons seek shelter within social networks and relocate to other towns and communities to join their families and clan members.

The performance of NEMA in managing the IDP crisis in Nigeria over the years has also come under intense scrutiny by stakeholders. A critical segment of the society; the Human Rights Watch (2018) contends NEMA has not performed optimally in the discharge of its statutory functions towards the displaced while NEMA claim to be doing its best relative to available resources (National Emergency Management Agency, 2018). NEMA's efforts towards addressing the plights of the IDPs in the views of Ajijola (2017) are grossly hampered by underfunding and lack of technical competence, while also noting that lack of awareness of available post-camp opportunities are part of the causal factors of victim's vulnerability to intense sufferings. The author suggests that NEMA should build a culture of curtailing mass displacement with an effective mechanism to identify all potential early warning internal displacement signals and address them before they burst in Nigeria.

What has been distilled from this review is that internally displaced persons are facing series of challenges from different crises; arising from the natural and human-induced crisis. The critical gap left for this study to fill borders on the fact that none of the existing literature has adequately studied the institutional capacity of NEMA to respond to the plights of the IDPs although the agency is the primary national responder to them, a gap this study intends to bridge by examining the response capacity of the agency to the burgeoning situation of the IDPs in North-eastern Nigeria.

## 4 Theoretical framework

This paper adopts the Structural Theory as its theoretical framework of analysis. The Structural theory as expanded by Rose (1998) and Paul (2003) explains the immediate and underlying factors which directly or indirectly lead to human displacement from their natural habitat. As noted in the theory, while political, economic, and social factors are critical factors responsible for the displacement of people, structural factors, including conflictive inter-group politics, lack of social justice, weak state institutions, and discriminatory political institutions, bitter religious and inter-tribal acrimonies and inter-group fragmentation are largely responsible for human displacement. Other structural factors for mass displacement include national security challenges, internal and cross-border criminality, and insurgency. The author also identified demographic factors such as environmental pollution, deforestation, drought, and natural disasters as key factors for human displacement. Other sources of demographic-induced displacement are overpopulation, natural disaster, weak economic opportunities, and non-integrated social institutions, which put pressure on human settlement. As noted by Rose (1998), some of these factors, especially violent political competition, natural calamities; such as famine and drought, inter-tribal acrimony, internal and cross border criminality, among others, often lead to forced displacement while socio-economic factors, especially weak employment opportunities and overpopulation could lead to voluntary displacement.

Arguing specifically on the structural politico-economy and power relations factors for insurgent induced internal displacement, Paul (2003) finds that there is a weak structural relationship between the government, the governed and socio-political and economic institutional structures put in place for achieving citizens' personal and group development aspirations. The author posits further that once conflict has amplified up to the crisis level between the government and the governed or among the governed, it often raises other social calamities, including the proliferation of arms, socio-economic dislocation, intense hardship, destruction of lives and property, the devastation of infrastructural facilities and human displacement, which leads further to human sufferings. And that the sustained conflict does prevent conflict victims, especially the internally displaced to get maximum relief assistance from disaster responders who are sometimes attacks by the belligerent fighters through land and air strikes leading to more epidemic disasters, including diseases because of overcrowded conditions in the IDPs camps.

The structural theory is apt and relevant for this study because the Boko Haram insurgency, which has led to the continued rise in the number of IDPs in North-eastern Nigeria, is a by-product of poor governance and religious extremism, which is largely unregulated by the government. The theory offers deep insight into the interlocking factors that sustain insurgency and IDPs in Northern Nigeria.

## 5 Methodology

This study made use of Key Informant Interview (KII), which is a qualitative research design that borders on the collection and analysis of non-numerical and non-experimental data to enhance a better understanding of concepts, opinions, and experiences (Dan, 2012; Pritha, 2020; Ashley, 2020). The use of KII as adopted in this study involved the administration of both structured and unstructured interviews to generate the required data from the key stakeholders in the management of the IDPs in North-eastern Nigeria. Those interviewed specifically were 166 respondents, consisting of 100 IDPs inmates, with 50 respondents in Borno, which has 70 percent of the IDPs, 30 respondents in Yobe State with 30 respondents from the IDPs inmates, and 20 respondents in Adamawa State. 50 IDPs management officials were also interviewed with 20 officials from the NEMA, 30 officials from the State Emergency Offices in Borno, Adamawa, and Yobe states, consisting of 10 from each state while 16 other camp commander were interviewed with 8 respondents from Borno, 5 from Yobe and 3 from Adamawa.

The use of the KII as adopted in this study allowed for the recording and preservation of the opinion of the respondents on tape, having been so permitted, which were re-played privately by the researchers for clarifications where necessary (Pritha, 2020). Though, the qualitative design as used in this study has the disadvantage of generating limited generalization because of the use of a relatively smaller sample size compared to the quantitative data (Dan, 2012), the advantage of focusing on smaller but relevant key informants, who are the primary stakeholders in the management of the IDPs crisis enables the generation of reliable data from the field (Dan, 2012). The use of KII also allowed the researchers to unveil the feelings, outer thought processes, and emotions of the respondents on the plights of the internally displaced persons and the response capacity of NEMA in North-eastern Nigeria. The qualitative research design was complemented by the use of secondary data, which was derived from books, journal articles, News tabloids, monographs, and previous academic projects on human disasters and refugee management. Data generated were descriptively analyzed.

The consents of the respondents under the qualitative research design were sought and obtained with permission granted to use their interview responses for this study but under the conditions that their names be unreferenced and unacknowledged; conditions, which the researchers adhered to.

## 6 Results

This section discusses the research findings on the plights of the IDPs, their coping strategies, and the response capacity of NEMA.

### 6.1 Plights of the IDPs

Findings from this study confirm that the IDPs in the North-eastern part of Nigeria are facing lots of challenges in their camps as they find it difficult to regain their pre-insurgent relatively better standard of living status before moving to the camp. Their pre-camp plights include tiredness and exhaustion from long journeys to the camp and psychological trauma from insurgent attacks, which sometimes cause stress disorder for some camp inmates. Some IDPs are also afflicted with extreme pains from injuries sustained when fleeing from Boko Haram to the camp while some others; especially pregnant women were confronted with forced labor and un-assisted childbirth on the road, which in some instances led to their death or that of the new infants. The mass displacement of persons by the insurgents has also curtailed IDPs' access to their farmland, markets, and other sources of livelihood, which eventually weakened their financial security even before getting to the camp, thus, forcing them into a life of misery in the camp.

Within the camp, the plights of most Internally Displaced Persons (IDPs) in North-Eastern Nigeria revolve around insufficient food, crowded accommodation, weak health care services, and insecurity, among others. In the area of food supply, lots of the IDPs are facing hunger and starvation in the camp as confirmed by them. A respondent, who stays in Bakkasi camp, which is the largest in Borno state; with IDPs mainly from Monguno, Gwoza, and Baga, confirmed that:

We have no enough food to eat here in the camp; we are always hungry because, in the past two months, the government people have not brought us food. As I am talking to you now, I and my kid siblings have not eaten since morning. We always eat our little breakfast late in the afternoon to be able to take us till the second day" (Key Informant Interview//Respondent/32/Female/Bakkasi IDP Camp//September 6//2019.

Despite the efforts of the governments, both at the federal and state levels to secure their camps, the IDPs are still facing security challenges, especially from suicide bombers. For instance, in September 2015, a Boko Haram suicide bomber attacked the IDPs Camps in Madagali and Yola, killing 12 persons. There was also the detonation of bombs inside a tent at an IDP camp in Gwoza, killing the bomber and two IDPs, the incidence of suicide bombing equally occurred at Bakassi camp in Maiduguri, Borno State in 2016. There were also cases of intra camp petty stealing and brutality, especially among children in the camp. Also, an Air Force fighter jet involved in counterinsurgency operations in North-Eastern Nigeria inadvertently fired at aid workers, security men, and displaced persons in error, killing no fewer than 100 persons on the IDP ground. An additional challenge to personal security in the camp is the vulnerability of the Internally Displaced Women to sexual abuse and other forms of Gender-Based Violence (SGBV). Though both male and female faces GBV in those camps, the female is often the systematic target of sexual violence by different stakeholders who manages the camp, including the security operatives, the camp management and people from the immediate communities where camps are located in the North-east. Some of the female inmates in the IDPs camp are lured by the stakeholders and strangers from outside with little financial token for sexual gratifications while there are incidences of outright rape in some instances. As posited by an IDP respondent:

It is bad enough that the internally displaced women and girls are not getting much-needed support for the horrific trauma they suffered at the hands of Boko Haram, It is even more disgraceful and outrageous that people who should protect women and girls are attacking and abusing them (Key Informant Interview/Female/Madagali, August 5/2019.

Shortage of accommodation is a major plight confronting the IDPs in North-eastern Nigeria. The Bakassi IDPs camp; one of the largest in Borno state, accommodates displaced persons mostly from Monguno, Gwosa, and Baga. The camp is not adequately fit for human shelter; being an uncompleted housing estate of the Borno State government, which was converted to camps for the IDPs in 2015 without adequate facilities such as light, water, and good roads. The United Nations High Commission on Refugees (UNHCR) also built 80 units of temporary shelters for the inmates, which bears their insignia. Due to a shortage of accommodation, many IDPs live in churches, mosques, town halls, abandoned and uncompleted buildings, and other forms of make-shift camps, which are grossly inadequate and unsuitable for accommodating the displaced population. To improvise for accommodation shortfalls, many IDPs made do with makeshift arrangements by gathering grass and sticks, which are fixed together on the ground in a circular shape and thatch the grasses on the sticks. The Healthcare delivery system is in shamble in many IDP camps as inmates are mostly supplied with mild pain relievers while serious cases are referred to major hospitals in town where many camp inmates lack money to pay for treatment.

### 6.2 Coping strategies within the IDPs camps

The coping strategies among the IDPs in the Northeast are very diverse. Their coping and survival strategies for reducing hunger include food rationing and food skipping. Food rationing entails the family's extension of breakfast consumption of both the raw and cooked food more than once with a small portion for each member of the family while many families sometimes eat once in a day by completely skipping breakfast, lunch, or dinner. Also, adults skip food in many instances to preserve sufficient rations for the children. Some IDPs take to begging for food or for money to buy food items. IDPs do sell off some of their raw foods to buy the required condiments to prepare soup while root flakes, especially the cassava flake "Garri" drink raw rather than been cooked. Due to acute food shortage in the camp, many inmates scavenge for food from eateries and wealthy homes with its attendant danger and concern for the health of the inmates over the possibility of consuming contaminated food. Some IDPs do hire motorcycles from their neighborhood resident owners to carry passengers, which enables them to get money for their basic needs while there is the practice of cultivating a small garden within the camp backyard to grow vegetables and other food condiments for family use. Some inmates, especially children, and the youth engage in outright stealing food from fellow inmates to survive while many women engage in prostitution as an opportunity to be fed directly or to get money to buy food for the family. Some of the children do offer their services as sale boys and girls in nearby markets and business areas with a token to take care of the family. Since security has largely relapsed in the camp, there is a joint inter neighborhoods watch to wade off criminals from outside but serious intra camp offenses are reported to the camp administrators by the security outfit. Owing to the high cost of buying drugs, many IDPs do not go to the hospitals but rely essentially on local medications from traditional herb sellers, which sometimes result in health complications, even though the herbs are very effective in many instances.

The IDPs in the Northeast also have inter and intra camp school Lessons in the absence of regular school attendance, which is largely coordinated by IDP members with higher educational attainment. Thus, it is very common to see secondary school students teaching those in primary schools while educated parents in the camp, especially mothers teach their children and those of their neighbors. There is regular inter and intra camp football competition among the youth to uplift their mood and promote inter and intra camp harmony. Some of the IDPs are trained as soap makers and cap, bag, and basket designers; they sell their products in the neighborhood market for survival. The youth hire barrow to carry goods for people on the market day while others fetch water for people to get money. In virtually all the camps in Maiduguri, every last Saturday of the month is declared as sanitation day reserved for the clean-up of the environment by the IDPs.

## 7 Response of NEMA to the plights of IDPs

Concerning the response of NEMA to the plights of the IDPs in North-eastern Nigeria, it is important to note that the rehabilitation and proper care for the IDPs is the primary responsibility of the concerned States but the scale and scope of the challenges in the area was overwhelming for the affected states to handle. Interestingly, the intervention of NEMA has relatively helped to ameliorate the suffering of the IDPs in the affected States as NEMA was documented to have carried out, in collaboration with the State Emergency Management Agencies within the North-east, the registrations of the IDPs in the camps, from where about two million inmates were registered as of 2019. The Agency also provided telephone facilities in the camps for IDPs to make contacts with their relatives for possible reintegration with missing relatives. To ameliorate the plight of camp inmates on hunger and food shortages, NEMA released Eight Hundred Million (N800, 000,000.00) Naira to supply food items to the IDPs while the distribution is ongoing. Part of the money was used by NEMA to purchase 200,000 bags of maize, 50,000 bags of rice and 250,000, a bag of millet for distribution to the IDPs camps in Borno, Yobe, and Adamawa, with Borno, allocated more than half of the food items because of a larger concentration of the IDPs in the state. To discourage food diversion in the camp, NEMA is now involved in the direct distribution of food items to the inmates as its officers move from shelter to shelter in the IDP camps to drop bags of grains for different households to share. The IDPs are given special meal tickets before they could get their food ration. To address the complaint of the inmate on inadequate food supply, NEMA has increased food rations per household of an average of 7 inhabitants from 4.5kg to 8.4kg. A key respondent who works with NEMA confirmed the increase in food ratio to the IDPs with the following statement:

We have critically assessed the food needs in the camp and the complaint of the inmates and what we have done is to double what we were earlier given to the IDPs. What we are giving them now is 8.4kg as against the 4.5kg they were collecting. The standard practice all over the world is 10.6kg so I think we are not doing badly in that regard" (Key Informant Interview, 2019).

Due to the synergy between NEMA and World Food Programs (WFP), the latter organization has also increased its food ration to 8.4kg in their own IDPs areas of food coverage. With the relative increase in the food supply, the narratives of many IDPs in the North-east have changed, as they confirm the changes in their diets. An IDP in the Bama camp said:

We are no longer hungry as before, we now have food. We have doctors here taking care of us, and food and clothes; we do not have any problems. They give us spaghetti for breakfast, rice, and beans for lunch and semolina for dinner we are grateful to God".

In the area of accommodation, NEMA constructed over 3,000 tents to bridge the shortfalls in the make-shift apartments offered to the inhabitants by state governments to accommodate more IDPs. The inhabitants were also provided with mattresses, buckets, and plates, among other household utensils. NEMA equally distributed clothing it got from non-governmental organizations to the IDP camps. The Aliko Dangote Foundation alone has built houses worth 2 billion for the orphaned and widowed IDPs, known as Dangote Village in Maiduguri. The housing estate consists of a set of 200 housing units with a school, hospital, irrigation farms, and poultry farms. Dangote also gave a cash donation of #100,000 for each of the inmates to start a new business after living in the camp and promise to pay the salary of the teachers in the Estate school for five years.

In the area of health care, NEMA has been providing Drugs to Major Hospitals in the Zone for the Treatment of Victims of Insurgency (TVI) free of charge. These hospitals include a Specialist Hospital in Yola, University of Maiduguri Teaching Hospital, and Nursing Homes in Maiduguri, Borno State Specialist Hospital and General Hospital, Potiskum. NEMA deployed ambulances at strategic locations to support and evacuate insurgency victims to hospitals from their camps or provide first Aid where necessary while the agency staffers also help in distributing the relief materials directly to the beneficiaries.

NEMA has also impacted positively on the amelioration of the plights of the IDPs by enthroning skill improvement programs to prepare them for post-camp resettlement by training them on relevant skills and providing them with working tools. For instance, sewing machines were provided to the IDPs with expertise in tailoring to sew clothes for fellow IDPs and the larger markets and get paid. The above findings confirm that NEMA has made credible input towards the amelioration of the plights of the IDPs with a reasonable measure of satisfaction.

## 8 Plights of the IDPs and response capacity of NEMA: interpretation and analysis of data

From the findings in this study, there is no doubt that the Boko Haram insurgency was primarily responsible for internal displacement in North-Eastern Nigeria. Other disasters-induced calamities in the area, including flooding, fire outbreak, rainstorm, windstorm, cerebra meningitis, Lassa fever, and cholera, harder-farmer and violent inter-communal conflicts also accounted for internal displacement. The causal factors for the sustenance of the Boko Haram phenomenon revolve around wrong religious indoctrinations, weak parental control, poor governance, manipulation of youth for political interests, and a high rate of youth unemployment. It is quite evident from this finding that to drastically reduce the plights of the IDPs in North-eastern Nigeria; the above causal factors need to be addressed. The study also reflected the dynamics of the displacement and the coping strategies of those displaced within the affected states in the northeast, which vary considerably. Thus, rural inhabitants are fleeing their villages and seeking refuge in the surrounding capitals of their local government areas and their State capitals in Yola, Maiduguri, and Damaturu while many displaced urban dwellers are seeking safety in Abuja; the Federal Capital Territory, and other state capitals across Nigeria. Though insurgency is largely responsible for the destruction of lives and property in North-Eastern parts of Nigeria, the factors that force people into refugee camps included the insurgent-induced state of insecurity in their natural habitats, which denied them livelihood and accommodation having destroyed their houses, ceaseless killings and monetary extortion of the populace by the insurgents, draconian implementation of religious laws and forceful conversion of people to Islam in the captured territories by the insurgents. Other factors for forceful dispersal of people to the refugee camp in the study area include high-handedness by the security Joint Task Force in counter-insurgency operations, the further threat of attacks of inhabitants by insurgents, and weak responses to insurgent attacks from the state security apparatus. This finding is a reflection of security lapses, especially in the rural areas where the insurgents sometimes operate for several hours without any meaningful resistance from the state security apparatus, which is evidence that the state security apparatus has no overwhelming capacity to curtail the insurgents; and an indication that it will take the IDPs longer time than expected to relocate back home. The withdrawal of the Chadian troops from the Multinational Joint Force, which opened their frontline areas of defense to easy attacks of the insurgents could continue to forced more people to flee to the IDPs camp for protection.

The worsening insurgent-induced food insecurity in North-Eastern Nigeria, which does not exclude the IDP camps, is indirectly responsible for the hunger being witnessed in the IDP camps. There is a food crisis in the northeast largely because of the worsening state of security in the region, leading to food insecurity for about 2 million IDPs. It is projected from these findings that food insecurity may likely affect another set of 5.2 million people who are not even internally displaced in North-eastern Nigeria but who could not farm optimally due to the state of insecurity on the farmland. More so, those who bring food from other parts of Nigeria to most North-eastern States for sales have tremendously reduced in number due to insecurity in the area, which could lead to worsening cases of food insecurity and low food intake related diseases such as malnutrition in the camps, which afflict inmates due to escalating cases of poor food consumption by the IDPs. Security concerns will also continue to contribute to hunger and food insecurity in North-eastern Nigeria. Within the geo-political zone, for instance, cultivation of major staple grains that are naturally taller than human beings (sorghum, maize, and millet) was restrained during the 2014 to 2019 farm seasons due to security intelligence report, which indicates that insurgents could hide in grain farms.

Apart from irregular supplies and shortages in the supply of food to the IDP camps, there are many cases of food pilfering and diversion by many stakeholders involved in the distribution of the food items. For instance, some officials of the Borno State Government were caught in the act of diverting IDPs' food to the markets. A Borno state high court later jailed two men for stealing and subsequently selling off 145 bags of rice meant for IDPs. The diversion of food and other relief items meant for the IDPs by the person charged with the responsibility of their distributions largely unabated, food insecurity among IDP inmates will likely worsen and increase hunger in camps.

To the response capacity of NEMA to the plights of the IDPs, it is evident that the intervention of NEMA has relatively helped to ameliorate the suffering of the IDPs in North-eastern Nigeria in collaboration with other relevant responders. Despite these relative achievements, the agency has been finding it increasingly difficult to manage the plethora of IDP crises due to logistic and financial problems that essentially borders on their large numbers and scarcity of resources to adequately fund the IDPs projects by NEMA. As indicated by NEMA (2019), over #2 billion is needed quarterly from the state and federal governments to take care of the IDPs in the three affected states out of which #483 million are made available quarterly for the purpose. NEMA sources much of its IDPs funding from non-governmental organizations but the economic recession, which was largely caused by the COVID19 pandemic in Nigeria, has dwindled funding by donor agencies. The above unfavorable trends leave NEMA in financial limbo most of the time to adequately care for the IDPs thus confirming the presumption that NEMA has no sufficient financial capacity to adequately ameliorate the sufferings of the IDPs.

The response of NEMA to the plights of the IDPs is also challenged by the weak coordinating capacity of the agency due to lack of effective coordination of the efforts of the numerous responders by NEMA for proper management of the IDPs. NEMA responders include local and international relief agencies, security agencies, and medical personnel but at a time, many of these responders encroach into NEMA's areas of responsibilities while abandoning their primary roles due to improper coordination of duties. For instance, the distribution of relief materials to the IDPs is the primary responsibility of NEMA officials; this responsibility is often hijacked by security personnel to enable them to divert the resources. Although NEMA has developed several response Plans to adequately respond to the plights of the IDPs in the North-East, these plans are rarely subjected to pilot tests, at least in one camp to confirm their efficacy and get responders acquainted with their roles, and internalize their standard operating procedures. NEMA is also bedeviled by training and logistic problems. Many of the responders are hardly trained on rescue operations. Thus, inadequate training of responders on search and rescue missions and other requisite skills are challenging to the success of NEMA in its task of responding to the plights of the IDPs. The current standard of NEMA's operational equipment is very low to respond adequately to the plights of the IDPs. The agency needs well-equipped mobility, including helicopters, vehicles, and communication gadgets, among others, for quicker responses to rescue operations in the camps, most especially in Gwoza and Konduga camps where the Boko Haram fighters still lay siege to attack relief workers and divert relief materials for their use.

Other critical factors that were responsible for weak responses of NEMA to the plights of the IDPs in North-eastern Nigeria revolve around the weak legal foundation and difficult operational terrains. Within the confines of legal ambit, the IDPs live within the borders of their own country while the responsibility for their protection and assistance rests on their national government, unlike the refugees whose protection is guaranteed in international law. However, due to the lack of precise and codified domestic laws to protect the IDPs in Nigeria, their protection has been incoherent, fragmented, and on an ad-hoc basis. These non-protective legal gaps have worsened the intractable position IDPs have found themselves as citizens as the capacity of NEMA to effectively manage internally displaced persons is mainly limited to providing short-term solutions on the humanitarian ground rather than legal.

## 9 Recommendations

The following recommendations are made to enhance the optimal response of NEMA to the plights of the IDPs in North-Eastern Nigeria. The National Emergency Management Agency should be well equipped, trained, and financed by the federal government to curtail the current challenges facing the IDPs in the camps. Federal and concerned state governments should as a matter of urgency intensify skill acquisition projects for internally displaced persons and empower them with the required funding to start their businesses.

The federal government should streamline the functions of all agencies working with NEMA and harmonize their operations for better coordination and optimal performances while stiffer penalties, including long term imprisonment, should be meted out to any responder caught diverting relief materials meant for the IDPs by the concerned authorities to serve as deterrence to others.

All the tiers of governments in the affected states and other stakeholders, including private sectors, international organizations, and civil society organizations should be more committed to alleviating the plight of the IDPs through improved funding.

Adequate implementation of the above recommendations could ameliorate the plights of internally displaced persons in North-eastern Nigeria.

## 10 Conclusion

The study concluded that the IDPs are facing series of challenges in North-eastern Nigeria, especially in the three states of Borno, Yobe, and Adamawa while the Boko Haram insurgency was the primary trigger of the mass internal displacement of persons in the area. Though NEMA has recorded some measure of achievements in managing the plights of the IDPs in the affected areas, the agency has not been able to perform these tasks optimally due to the protracted nature of the insurgency and limited funding of its operations. The agency is also incapacitated by legal, coordinating, logistics, and operational inhibitions. The Study concluded that NEMA currently has the minimal operational capacity to ameliorate the plights of the IDPs in North-Eastern Nigeria.

## Declarations

### Author contribution statement

Raji Shittu: Conceived and designed the experiments; Performed the experiments; Analyzed and interpreted the data; Contributed reagents, materials, analysis tools or data; Wrote the paper.

Adekayaoja Folashade Arinola: Conceived and designed the experiments; Performed the experiments; Analyzed and interpreted the data; Contributed reagents, materials, analysis tools or data; Wrote the paper.

Emmanuel Agaku: Conceived and designed the experiments; Performed the experiments; Analyzed and interpreted the data; Contributed reagents, materials, analysis tools or data; Wrote the paper.

Akujobi James: Conceived and designed the experiments; Performed the experiments; Analyzed and interpreted the data; Contributed reagents, materials, analysis tools or data; Wrote the paper.

Hamzat Ade Ayinde: Conceived and designed the experiments; Performed the experiments; Analyzed and interpreted the data; Contributed reagents, materials, analysis tools or data; Wrote the paper.

### Funding statement

This research did not receive any specific grant from funding agencies in the public, commercial, or not-for-profit sectors.

### Data availability statement

Data referenced in article.

### Declaration of interests statement

The authors declare no conflict of interest.

### Additional information

No additional information is available for this paper.
